# Pilomatrixoma Arising Juxtaposed to Congenital Melanocytic Nevi: Concern for Malignant Degeneration? A Previously Unreported Association

**Published:** 2016-07-22

**Authors:** Carol. E Soteropulos, Paschalia M. Mountziaris, Oluwaseun A. Adetayo

**Affiliations:** Division of Plastic and Reconstructive Surgery, Albany Medical Center, Albany, NY

**Keywords:** pilomatrixoma, pilomatricoma, congenital melanocytic nevus, congenital nevus, skin lesion

## DESCRIPTION

We present a 13-year-old male patient with congenital melanocytic nevi and a yearlong history of enlarging right preauricular mass. Examination revealed 2 congenital melanocytic nevi overlying a large, firm, mobile 2.7 × 2.5-cm subcutaneous mass with irregular borders. Pathology confirmed intradermal melanocytic nevi; the underlying mass was a calcified and ossified pilomatrixoma.

## QUESTIONS

1. **What is a pilomatrixoma/pilomatricoma?**
2. **What are the causes and syndromic associations of pilomatrixomas?**
3. **What are congenital melanocytic nevi, and what is the risk of malignant transformation?**
4. **What is the association between pilomatrixomas and congenital melanocytic nevi?**

## DISCUSSION

Pilomatrixomas are cutaneous adnexal tumors, also referred to as calcifying epithelioma of Malherbe, as it was first described by Malherbe and Chenantais more than 100 years ago.^1^^,^^2^ Although they can occur at any age, they most often present in the first and sixth decades of life in a bimodal distribution.^3^ They arise from the lower portion of the hair follicle matrix cells and can contain areas of calcification and ossification.^3^ These tumors frequently arise in the head and neck region, presenting as slow-growing, firm masses, which can be deep or superficial, and involve ulceration through the skin.^2^^-^4

Pilomatrixoma has been reported to arise in a site of trauma or previous infection.^4^ Although usually they are singular lesions, multiple tumors have been reported in the literature.^3^ The finding of multiple pilomatrixomas in a single patient should warrant investigation for genetic disorders including Gardner syndrome, myotonic dystrophy, xeroderma pigmentosum, and basal cell nevus syndrome.^1^^,^^4^^,^^5^ Cases of malignant transformation have been reported most often in the adult population, although pilomatrixoma is not characterized as a commonly malignant tumor.^1^^,^^3^ Most often the diagnosis is clinical, treatment is complete excision, and recurrence is rare.^1^^,^^4^

Congenital melanocytic nevi are benign proliferations of the nevus cell type of melanocytes that present at birth or within the first few months of life. They can extend into the dermis and subcutaneous structures and are usually small (<1.5 cm) or medium sized (1.5-20 cm).^6^ The small- and medium-sized nevi are rarely malignant, with the risk of transformation to melanoma under 1% in a lifetime.^6^

Although many case series have been published on the topic of pilomatrixoma, the association between pilomatrixoma and intradermal melanocytic nevus has not yet been reported. There have been studies detailing a pigmented type of pilomatrixoma, in which the presence of melanin within the pilomatricoma itself was the distinguishing feature.^7^^,^^8^ One case report described a child with multiple large pilomatrixomas arising near diffuse epidermal nevi in the absence of an epidermal nevus syndrome.^6^

**Figure 1:**
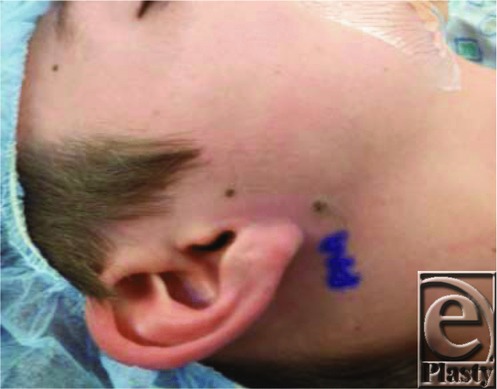
Preoperative photograph showing the overlying melanocytic nevi.

**Figure 2:**
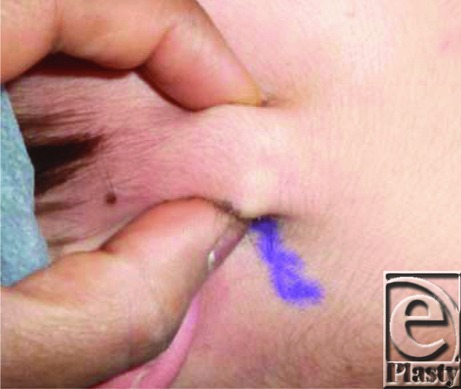
Preoperative photograph demonstrating the size and location of the enlarging mass.

**Figure 3:**
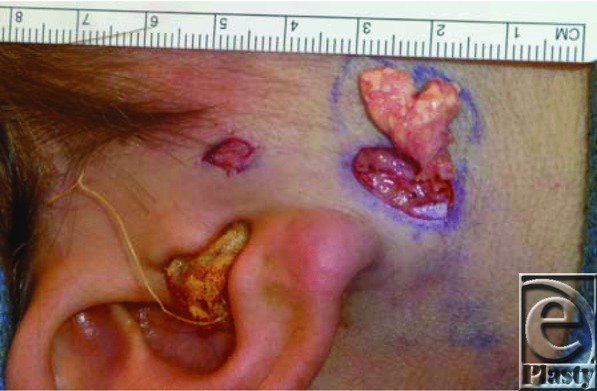
Intraoperative photograph showing the excised pilomatrixoma and marking pen outlining the dimensions of the lesion.

We present a unique case of an otherwise healthy 13-year-old male patient with 2 melanocytic nevi (Fig 1) overlying an enlarging mass (Fig 2), which raised concern for malignant degeneration and prompted referral for consultation. The patient underwent an excisional biopsy. The operative and pathologic findings were consistent with a pilomatrixoma, an association that has not been previously reported in the literature. Although it is possible that these lesions were separate entities, another interesting finding was that there was only a very thin fascial plane separating the 2 superficial nevi from the underlying 2.7 × 2.5-cm pilomatrixoma (Fig 3). The larger of the 2 nevi was firmly adherent to the dermis over the superior aspect and the underlying calcified mass inferiorly. The pathology report confirmed overlying intradermal melanocytic nevi and underlying calcified and ossified pilomatrixoma. The pilomatrixoma contained islands of bone, with ghost cells surrounded by fibrosis, regions of melanization, and foreign body giant cells. The patient did not have a history of trauma or infection in the area where the pilomatrixoma was located. This case may be a sporadic association between the 2 entities or represent a shared etiology yet undescribed.

## References

[1] Hassan SF, Stephens E, Fallon SC (2013). Characterizing pilomatricomas in children: a single institution experience. J Pediatr Surg.

[2] Duflo S, Nicollas R, Roman S, Magalon G, Triglia JM (1998). Pilomatrixoma of the head and neck in children: a study of 38 cases and a review of the literature. Arch Otolaryngol Head Neck Surg.

[3] Julian CG, Bowers PW (1998). A clinical review of 209 pilomatricomas. J Am Acad Dermatol.

[4] Pirouzmanesh A, Reinisch JF, Gonzalez-Gomez I, Smith EM, Meara JG (2003). Pilomatrixoma: a review of 346 cases. Plast Reconstr Surg.

[5] Yoshimoto S, Ichinose M, Udagawa A, Matsumoto H, Shimizu S, Danino AM (2002). Are multiple pilomatricomas rare?. Plast Reconstr Surg.

[6] Johnson LM, Newell B (2011). Multiple large pilomatricomas in the setting of diffuse epidermal nevi. Pediatr Dermatol.

[7] Ishida M, Okabe H (2013). Pigmented pilomatricoma: an underrecognized variant. Int J Clin Exp Pathol.

[8] Tallon B, Cerroni L (2010). Where pigmented pilomatricoma and melanocytic matricoma collide. Am J Dermatopathol.

